# MRI‐based polymer gel dosimetry for validating plans with multiple matrices in Gamma Knife stereotactic radiosurgery

**DOI:** 10.1120/jacmp.v12i2.3333

**Published:** 2011-01-31

**Authors:** N. Gopishankar, Yoichi Watanabe, Vivekanandhan Subbiah

**Affiliations:** ^1^ Gammaknife Unit, Neurosciences Centre All India Institute of Medical Sciences New Delhi India; ^2^ Department of Therapeutic Radiology University of Minnesota Minneapolis MN USA; ^3^ Neurobiochemistry, Neurosciences Centre All India Institute of Medical Sciences New Delhi India

**Keywords:** Gamma Knife, multiple matrices, polymer gel dosimetry, 3D dose distribution

## Abstract

One of treatment planning techniques with Leksell GammaPlan (LGP) for Gamma Knife stereotactic radiosurgery (GKSRS) uses multiple matrices with multiple dose prescriptions. Computational complexity increases when shots are placed in multiple matrices with different grid sizes. Hence, the experimental validation of LGP calculated dose distributions is needed for those cases. For the current study, we used BANG3 polymer gel contained in a head‐sized glass bottle to simulate the entire treatment process of GKSRS. A treatment plan with three 18 mm shots and one 8 mm shot in separate matrices was created with LGP. The prescribed maximum dose was 8 Gy to three shots and 16 Gy to one of the 18 mm shots. The 3D dose distribution recorded in the gel dosimeter was read using a Siemens 3T MRI scanner. The scanning parameters of a CPMG pulse sequence with 32 equidistant echoes were as follows: TR=7s, echo step = 13.6 ms, field‐of‐view = 256 mm× 256 mm, and pixel size=1 mm×1 mm. Interleaved acquisition mode was used to obtain 15 to 45 2‐mm‐thick slices. Using a calibration relationship between absorbed dose and the spin‐spin relaxation rate (R2), we converted R2 images to dose images. MATLAB‐based in‐house programs were used for R2 estimation and dose comparison. Gamma‐index analysis for the 3D data showed gamma values less than unity for 86% of the voxels. Through this study we accomplished the first application of polymer gel dosimetry for a true comparison between measured 3D dose distributions and LGP calculations for plans using multiple matrices for multiple targets.

PACS number: 87.53.Ly, 87.55‐x, 87.56 ‐g

## I. INTRODUCTION

Stereotactic irradiation with Leksell Gamma Knife (Elekta Instrument AB, Stockholm, Sweden) is one of primary methods used for the stereotactic radiosurgery of intracranial lesions. This technique is a well established option to radiosurgical treatment of arteriovenous malformations, benign and malignant tumors, and functional disorders.^(1)^ It consists of an array of cobalt‐60 sources that naturally emit gamma ray photons at a predictable rate. The 201 separate beams allow for a very precise delivery of radiation to a specified volume without severely damaging surrounding brain tissue. Single or multiple isocenters (shots) with four available helmets (4, 8, 14, and 18 mm beam diameters) can be used to treat targets of any shape. The dose is prescribed to the periphery at 50% isodose level in most cases, although lower prescription below 50% and higher prescription isodoses up to 100% can also be used in some cases. For a treatment, the Leksell stereotactic frame is attached to a patient's head under local anesthesia to establish a three‐dimensional (3D) coordinate system for the precise determination of target location through imaging usually with magnetic resonance imaging (MRI) scanners. The use of MRI as the primary imaging modality in radiosurgery has become increasingly attractive because of its excellent depiction of the brain's normal anatomy and demonstration of lesions not seen with other imaging modalities.

The excellent treatment delivery accuracy of Gamma Knife stereotactic radiosurgery (GKSRS) stems from the increased accuracy and precision of each individual step in the entire treatment procedure, which includes imaging, target localization, treatment planning, dose calculations and patient positioning, in addition to high mechanical accuracy and precise dose delivery. To assure the quality of the whole treatment procedure, an exhaustive quality control program is required. Since GKSRS can create rather complicated 3D dose distributions, the dosimetric system should be able to measure the 3D dose distributions. Polymer gel dosimeter can satisfy this requirement. There are many studies on applications of the polymer gel dosimetry technique to GKSRS.^(^ ^2^ ^–^ ^12^ ^)^ However, most of these studies evaluated the 3D dose distributions computed by the Leksell GammaPlan treatment planning system (LGP) (Elekta Instrument AB, Stockholm, Sweden) for cases with single shot or multiple shots placed in one dose matrix. There are three other cases for which dosimetric verification is needed because there are non‐trivial differences in the computational algorithms for these more complex cases involving multiple matrices, plugging and tissue heterogeneity. Multiple matrix‐based planning is one of the unique planning methods in which several dose matrices are combined to produce isodose distributions for cases having more than one tumor that are widely spread. This method also applies to situations where the tumor is too large to be covered in a single matrix. A single dose matrix consists of 31×31×31 grid points. Hence, even with the largest grid size available (or 2.5 mm), the largest volume covered by a single matrix is 465.48 cm^3^. Because the computational algorithm handling this case is different from the algorithms for the cases using only one matrix, experimental verification is urgently needed. An important question is the validity of the multiple matrix method, in particular, with older versions of LGP, in terms of the dose prescription to multiple targets scattered over the multiple matrices. To answer this question, Sandilos et al.^(9)^ recently used polymer gel dosimetry. In their study, dose delivery accuracy was evaluated by comparing measured dose distributions with corresponding calculations. A digitization method was used for selecting specific isodose lines for comparison purpose. A comprehensive dose comparison for the whole 3D volume was not shown. Here, we present the evaluation of the LGP dose calculation accuracy for multiple matrix cases using the MRI‐based polymer gel dosimetry technique. For this study, the dose data of multiple matrices extracted from LGP were numerically combined into one matrix using an in‐house program at the same positions as they were present in LGP. The resulting dose matrix (named as global matrix) was used for comparison with measured dose distributions.

## II. MATERIALS AND METHODS

### A. Polymer gel phantom

**Figure 1:**
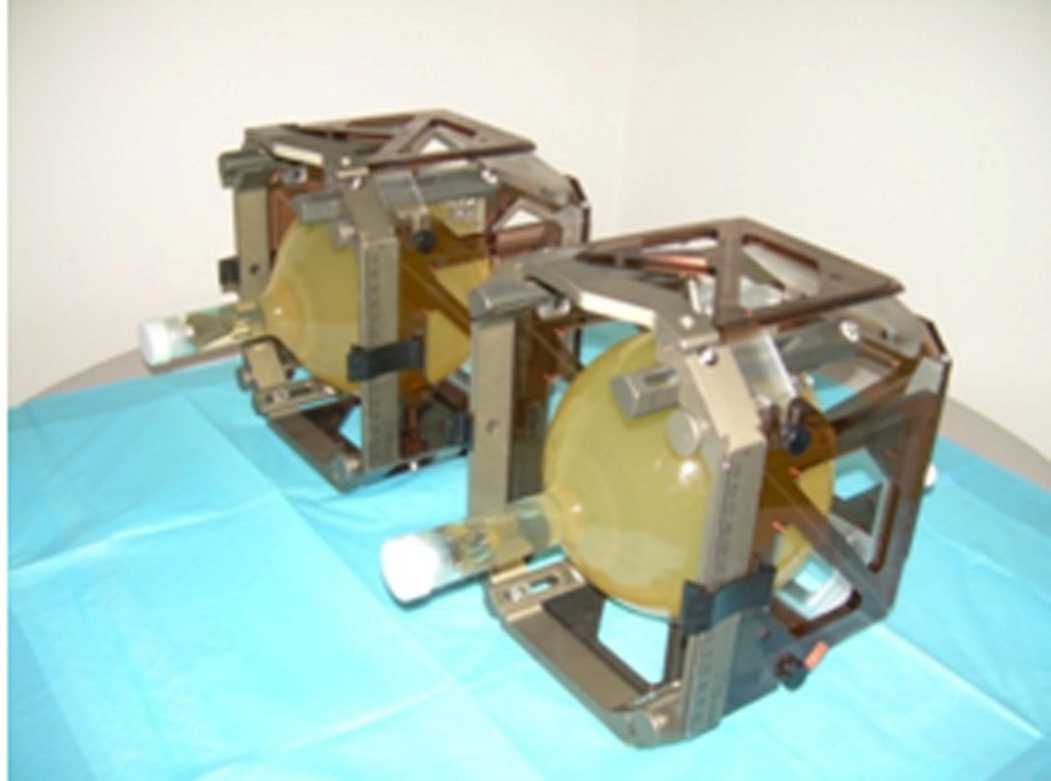
Phantoms fixed in Leksell frame with indicator box for MRI scanning.

BANG3 polymer gel was purchased from MGS Research Inc. (Madison, CT) for this study.^(13)^ The spin‐spin relaxation rate (R2) of the water protons in this gel increases with absorbed dose, independent of dose rate or radiation quality. BANG3 polymer gel contained in a head‐sized glass bottle simulated the entire treatment process of GKSRS. It was shown before that using a large polymer gel phantom mimicking a human head creates a uniform medium and hence reduces measurement uncertainties.^(8)^ Two 16‐cm diameter spherical phantoms filled with BANG3 were fixed in the Leksell stereotactic frame using a set of four vinyl suction cups. (See Fig. 1 for the phantom setup.)

### B. MRI scanning

For R2 measurement, we used a multispin‐echo pulse sequence of Carr‐Purcell‐Meiboom‐Gill (CPMG)^(14)^ with 32 equidistant echoes. Other imaging parameters were TR=7 s, echo step=13.6 ms, pixel size=1 mm×1 mm. Interleaved acquisition mode was used to obtain 15 to 45 2‐mm‐thick slices. The gel phantoms were scanned with a MAGNETOM Trio, a Tim 3T MRI scanner (Siemens Medical Solutions, Erlangen, Germany).

### C. Treatment planning

For a single target case, Leksell GammaPlan (LGP) treatment planning software, Version 5.6 and 8.2 (Elekta AB, Stockholm, Sweden), uses a cubic matrix of 31×31×31 grid points with variable grid spacing for 3D dose calculations. For multiple target treatments where targets are usually separated from each other and cannot be included in the same matrix or different prescription doses have to be ascribed to different targets, dose is calculated in multiple matrices. In a single plan we normally create as many as 10 to 12 matrices for clinical treatments.

**Figure 2:**
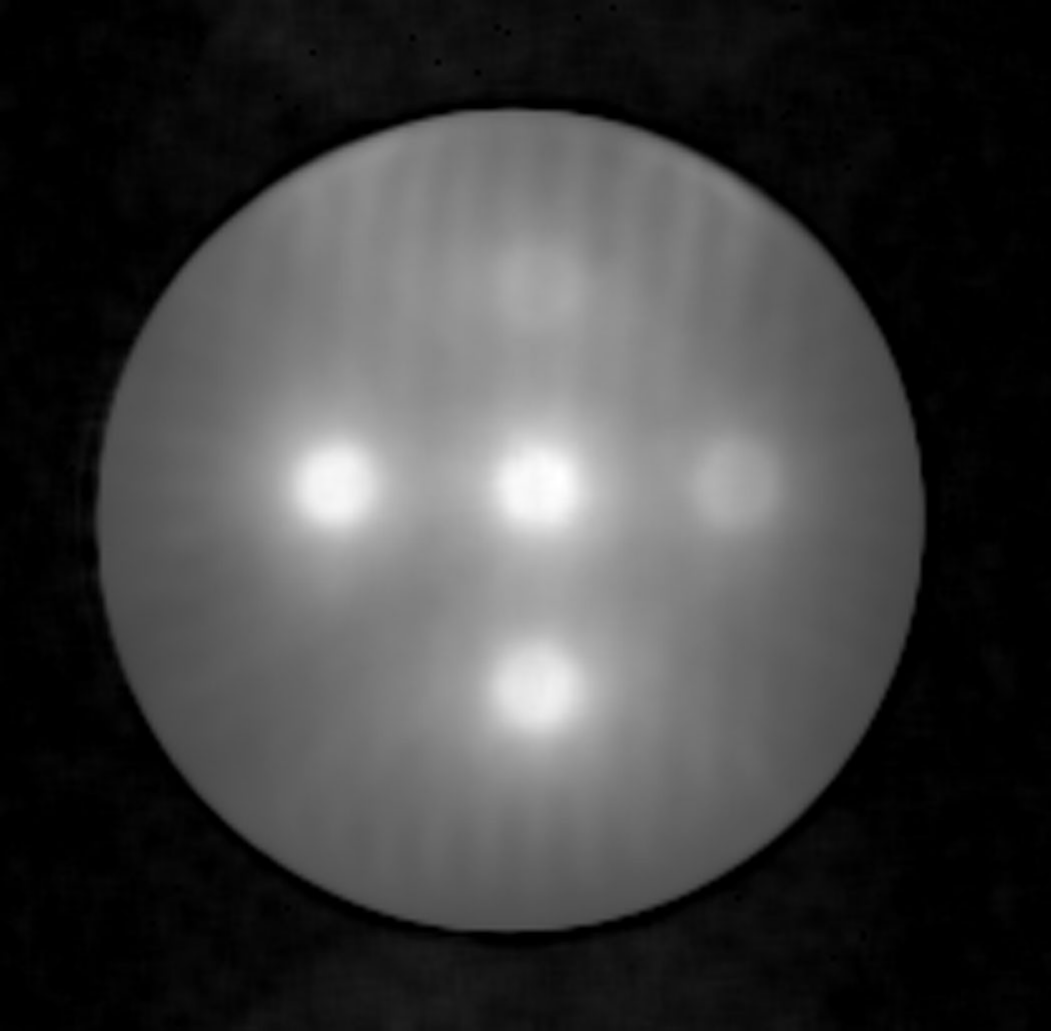
T2‐weighted image of phantom A with five 18 mm collimator shots delivering five different doses. The maximum dose to the shot at the center was 16 Gy. The maximum doses to the peripheral shots were 3, 5, 8, and 12 Gy.

For this study, the calibration of the BANG3 polymer gel was performed using five 18 mm collimator shots placed on single transverse plane (phantom A). Those shots delivered maximum doses of 3, 5, 8, 12, and 16 Gy. We used the 18 mm shot for gel calibration because the machine output was calibrated with this collimator size. Figure 2 shows the shot positions in a T2‐weighted MR image. A treatment plan simulating a multiple matrix case was generated with three 18 mm shots and one 8 mm shot (phantom B). The details of the shots used for the phantom B irradiation are summarized in Table 1.

**Table 1:** Summary of the dose plan created for irradiation in Phantom B.

*Matrix*	*Shot No*	*X (mm)*	*Y (mm)*	*Z (mm)*	*Gamma Angle (deg)*	*Prescription dose (Gy) at 50% Isodose*	*Max (Gy)*	*Collimator (mm)*	*Grid Size (mm)*
A	1	80	110	100	90	8.0	16.0	18	1.6
B	2	110	90	100	90	4.0	8.0	18	1.6
C	3	80	70	100	90	4.0	8.1	8	1.0
D	4	80	110	64	90	4.0	8.0	18	1.6

### D. Irradiation procedures

Two phantoms (phantoms A and B) were used for this study. A Leksell stereotactic frame was attached to the phantom by fastening four fixation screws so that the reference coordinate system defined by fiducial markers lays aligned with the stereotactic coordinate system of the Gamma Knife unit (Model 4C). The depth to the unit centerpoint (UCP) was measured in the similar fashion to that of a patient. The phantom was scanned before irradiation using a Siemens 3T MRI scanner with the FLASH pulse sequence, which is also used for GKSRS patient imaging. The acquired images were transferred to LGP for treatment planning. The gel phantom was stored overnight in the Gamma Knife suite to reach thermal equilibrium before irradiation. Next day, the gel phantom (already mounted in the stereotactic frame) was set on the Leksell Gamma Knife unit. The phantom was irradiated based on the LGP plan using the automatic positioning system.

### E. Analysis methods

We computed R2 values by assuming an exponential decay of the echo signal using an in‐house MATLAB (The MathWorks Inc., Natick, MA) program. The maximum likelihood estimator was used to estimate the R2 values. We obtained a calibration relationship between R2 and absorbed dose using the data from phantom A. The R2 images of phantom B were converted to dose images by applying the calibration equation.

**Figure 3:**
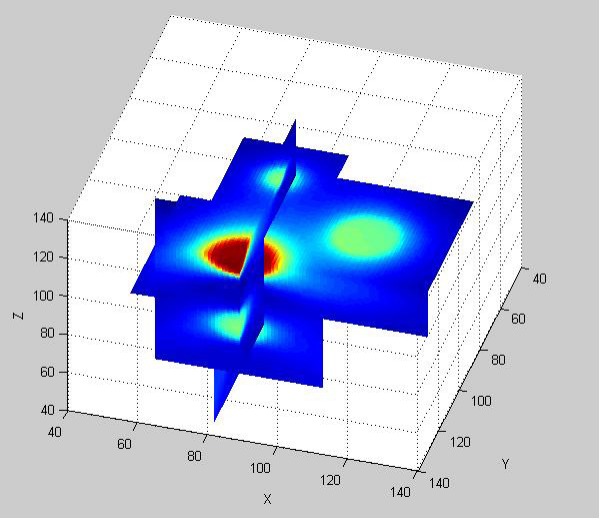
Dose distributions plotted on three orthogonal planes for the calculated dose data stored in the global matrix of phantom B.

The LGP software saves calculated dose data in a 3D matrix form with 31×31×31 elements. For phantom B, we used four such matrices for four individual shots. To compare measured dose distributions with LGP calculated dose distributions, the calculated data in multiple matrices had to be converted into a single matrix. A MATLAB program was used to combine the calculated dose data from separate matrices into a single matrix, called global matrix. The grid size of the global matrix was chosen as the smallest grid size among the four matrices. A 3D illustration of four shots shown in Fig. 3 was obtained by using the global matrix generated for phantom B.

The global matrix size for phantom B was 80×79×85 with voxel size of 1×1×1 mm^3^. The measured gel dose was represented as a 256×256×45 matrix. The voxel size was 1×1×2 mm^3^. The measured dose matrix was converted to a matrix whose elements are the doses at the points corresponding to the grid points of the LGP dose matrix. A 3D linear interpolation method (the “interp3” routine in MATLAB) was used for this conversion.

A MATLAB graphics user interface (GUI) program named GK3DCMP was developed to perform 3D comparison between the experimental data and the LGP calculated data. The dose comparisons were accomplished with several methods. The voxel‐by‐voxel dose difference values were grouped into dose bins based on the dose difference and the LGP calculated dose. Then, for 3D dose comparison, we generated a dose volume histogram (DVH), a differential dose volume histogram (DDVH), a dose‐dependent dose‐difference diagram (D4), and a dose difference histogram (DDH).^(8)^ Dose comparisons were also made by overlaying two isodose distributions on several transverse planes, as well as on coronal and sagittal planes. Note that, for this study, the dose difference was defined as the percentage difference between the calculated dose and the measured dose. The gamma indices^(15)^ in 3D space were computed using a relatively fast computational method similar to the recently published method.^(16)^ As the tolerances for the gamma‐index calculation we chose 3% dose difference and 3 mm distance‐to‐agreement.

## III. RESULTS

**Figure 4:**
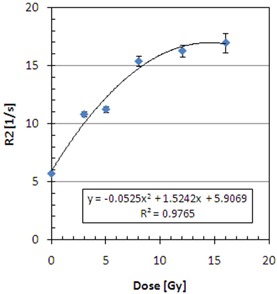
Calibration curve used to convert R2 into dose.

Figure 4 shows the experimental data for the dose‐response characteristics of the BANG3 polymer gel. It indicates that the dose response of the current polymer gel was non‐linear for doses higher than 10 Gy. A second degree polynomial fit was made for the calibration data and it was used for converting R2 into absorbed dose. Because of the sparseness and scattered nature of the calibration data, the coefficient of determination R2 was relatively small (i.e., 0.9765).

**Figure 5:**
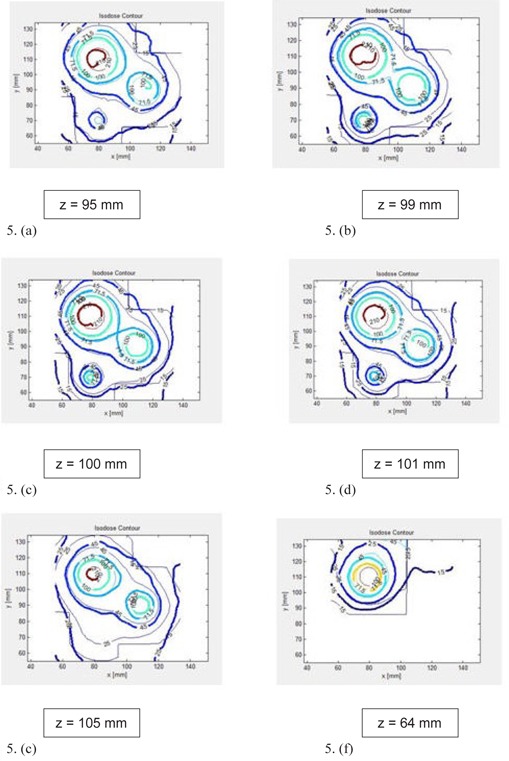
Comparison of BANG3 measured (solid line) and LGP calculated (thin line) dose distributions plotted on transverse planes: (a) Z=95 mm, (b) Z=99 mm, (c) Z=100 mm, (d) Z=101 mm, (e) Z=105 mm, (f) Z=64 mm. The labels of isodose curves indicate the percentage dose normalized to 7.2 Gy; or 100 indicates 7.2 Gy.

Figures 5 (a) to (e) present the measured and calculated dose distributions on transverse planes for different Z positions (i.e., Z=95,99,100,101 and 105 mm). Note that in these figures, the index values of isodose lines were calculated so that the maximum delivered dose of 16 Gy corresponds to 227.5, which was the maximum value in the global dose matrix for this case. All these figures indicate that 71.5 (5.03 Gy) and 100 (7.03 Gy) isodose lines agree within a 1 mm distance between measurements (thick lines) and LGP calculations (thin lines).

In Figs. 5 (a) to (e), the shot at the top left is the 18 mm shot with the maximum dose of 16 Gy (referred to as shot #1 in Table 1) and the shot at the middle right is the 18 mm shot with the maximum dose of 8 Gy (shot #2 in Table 1). The isodose lines of those two shots are separated for higher doses (e.g., isodose line 100 (7.03 Gy)). Lower isodose lines of those shots are continuous (e.g., isodose line 45 (3.6 Gy)). The isodose lines of those two shots contact one another at one point in 3D space for a dose level of 71.5 (5.03 Gy). Figure 5 (c) clearly shows such a point. We can call this point “reconnection point”. Note that the reconnection points of measured and calculated dose distributions spatially coincide, as seen in the figure.

Figure 5 (f) shows the isodose lines on Z=64 mm transverse plane, where an 18 mm shot with the maximum dose of 8 Gy (shot #4 in Table 1) was placed. The isodose comparison showed more than 5 mm deviation between measured and calculated doses.

**Figure 6:**
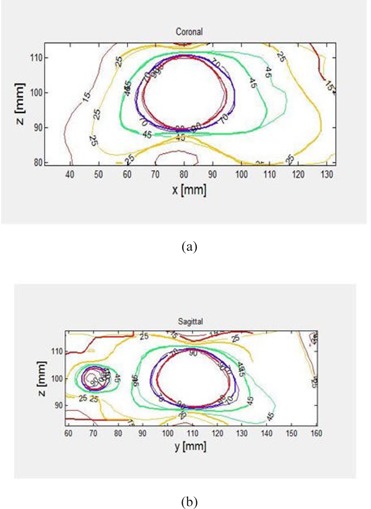
Comparison of BANG3 measured (solid line) and LGP calculated (thin line) dose distributions: (a) coronal plane at Y=110 mm, showing shot #1; (b) sagittal plane at X=80 mm, showing shot # 1 (right) and #3 (left).

The dose distributions on the coronal and sagittal planes presented in (Figs. 6a) and (b) show clear differences between measurements and calculations at isodose levels of 15 (1.2 Gy), 25 (2.0 Gy), and 45 (3.6 Gy), whereas there was good agreement at isodose levels of 70 (5.6 Gy) and 90 (6.5 Gy).

The normalized peak doses of the five shots were measured to be 100.0%, 48.2%, 58.2% and 36.4% for shots in matrices A, B, C, and D, respectively. Note that the expected peak doses for shots #2, #3, and #4 were 50% (or 8 Gy) of the shot #1 (or 16 Gy). Hence, the measured peak doses for shots #3 and #4 were different from the expected values by a large amount.

**Figure 7:**
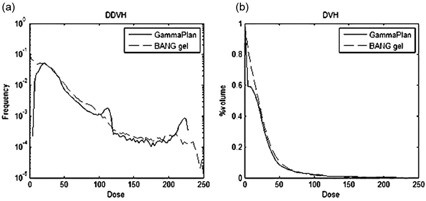
Dose‐volume comparisons of measurements (solid line) and LGP calculations (thin line): (a) DDVH; (b) DVH. The value of 227.5 in the horizontal axis corresponds to 16 Gy.

Figure 7 shows the differential dose volume histogram (DDVH) and the dose volume histogram (DVH) for calculated and measured dose data. Remember that the maximum of the calculated dose (16 Gy) was set to 227.5 in this figure. The figure shows a good overall agreement between two methods. However, there were two peaks corresponding to 8 and 16 Gy appearing in the DDVH curve of the calculated dose, whereas the curve for the measurement does not show those spikes. There are sharp peaks with the DDVH curve of the calculated dose because for an 18 mm diameter shot there is a constant dose volume of the maximum dose (i.e., 8 or 16 Gy) around the center of the shot position. But, the polymer gel dosimetry could not reproduce those relatively constant high‐dose volumes.

To analyze the differences in measured and computed doses in more detail, we now focus on a subspace of the entire measurement volume. We chose a subvolume, which included three shots (shots #1 to #3) positioned on the Z=100 mm transverse plane as shown in Fig. 5(c). The subvolume was a parallelepiped volume ranging from 70 mm to 110 mm, 70 mm to 120 mm, and 90 mm to 110 mm, in X, Y, and Z directions, respectively.

**Figure 8:**
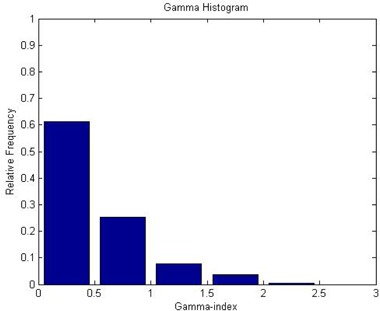
Histogram showing the Gamma index distribution. The criteria for the gamma‐index calculations were 3% dose difference and 3 mm distance‐to‐agreement. The calculation volume was limited to a subspace: x=70 to 110, y=70 to 120, and z=90 to 110.

First, the gamma‐index analysis was performed for this subvolume. The result is summarized as a histogram in Fig. 8, which shows that 86% of voxels included in the gamma‐index calculations had the value smaller than unity.

**Figure 9:**
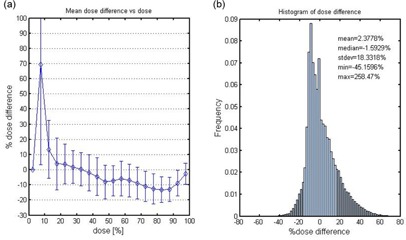
Dose‐dependent‐dose‐difference‐diagram (D4 diagram) (a), with the diamond symbols indicating the mean dose difference and the error bars indicating one standard deviation; dose‐difference histogram (DDH) (b), a frequency histogram of dose difference representing the mean, median, standard deviation, minimum and maximum dose difference between the measured and calculated dose.

For the same subvolume, the dose comparison was done using a dose‐dependent dose‐difference diagram (D4) and a dose difference histogram (DDH). We calculated the percentage dose difference ratios. The D4 diagram in Fig. 9(a) shows the mean dose difference (indicated by the diamond symbols) as a function of the dose. The error bars are for one standard deviation. In the figure, the 100% dose corresponds to 16 Gy. This figure shows that the dose difference varied within ± 10% for the dose greater than the 10% dose level (or 1.6 Gy). Larger deviations were observed for a lower dose range of below 1.6 Gy. From the DDH given in Fig. 9 (b), we found that the mean difference between the measured and the calculated doses was 2.4%± 18.3%.

## IV. DISCUSSION

For calibration, the desirable characteristic of any dosimeter is to have a linear response between absorbed dose and a physically measurable quantity, to which the dose is related.^(17)^ It is known that the polymer gels like BANG3 exhibit a saturation behavior for large doses.^(17)^ In fact, the calibration equation for this study had to be represented by a quadratic function. Furthermore, it is noted that the measured dose had to be multiplied by a scale factor to best match the calculated dose for the current study. Therefore, the nonlinearity of the calibration equation potentially resulted in incorrect doses after the rescaling operation.^(17)^

There were only six calibration points used for deriving the calibration equation, and the coefficient of determination for the curve fitting was relatively small (i.e., 0.976). This yielded very large uncertainty in the estimated coefficients in the quadratic calibration equation. The statistical uncertainty of the estimate R2 values was estimated to be in the order of 2% or less. When those uncertainties are taken into account in the uncertainly propagation equation,^(^ ^17^ ^,^ ^18^ ^)^ the uncertainty of the dose obtained using this calibration equation was inherently large, as large as 100%. This can partially explain the observed large differences between the measured and calculated doses, in particular at lower dose levels.

One example of the shortcomings with the calibration equation we used for this study is recognizable with the DDVH curves in Fig. 7(a), where the measured data failed to reproduce the two peaks, each corresponding to the 8 Gy and 16 Gy volumes of the flat regions in the dose volumes generated by the 18 mm shots. The disappearance of the 8 Gy peak may be due to the 2 mm spatial resolution in the Z‐direction for the measurements. The saturated region in the dose‐response calibration relation is poorly represented by a quadratic equation. Hence, the use of this equation results in overestimation of the dose greater than 16 Gy. This appears as the measurement curve extending beyond the 227.5 (or 16 Gy) value.

As we discussed in the Methods and Materials section above, the voxel sizes for LGP dose calculations of phantom B were 1.6×1.6×1.6 mm^3^ for the matrices with 18 mm shots and 1.0×1.0×1.0 mm^3^ for the matrix with an 8 mm shot (see Table 1). These matrix data were combined to create a global matrix with a 1.0×1.0×1.0 mm^3^ grid size for dose comparisons. Meanwhile, the voxel size was 1.0×1.0×2.0 mm^3^ for the measured dose. The calculated and measured 3D dose distributions were compared by interpolating the calculated and measured doses in a common 3D space with 1.0×1.0×1.0 mm^3^ grids. The linear interpolation in 3D was performed using a numerical routine of MATLAB. These interpolation processes may cause some errors in the comparison.

**Figure 10:**
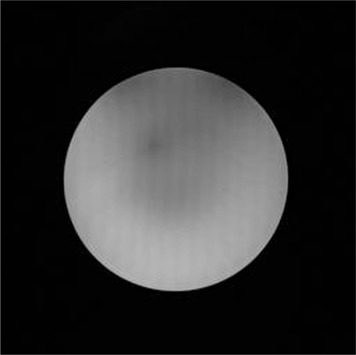
An spin‐echo image of phantom B presenting the artifacts. The image was acquired at echo time of 54.4 ms for slice number 23 (out of 45 slices). The two dark areas at the top edge of the phantom indicate the susceptibility artifacts due to the metallic fixation pins. We can also observe the stripes running in the top‐to‐bottom direction.

Artifacts such as signal inhomogeneity and rings were observed in the images of our BANG3 gel phantoms, as seen in Figure 10. Figs. 5 (a) to (f), which indicate that the 3.6 Gy isodose curves (denoted by 45) do not agree each other near the upper edge of the plots. One of reasons for the disagreement is the metal artifacts caused by the fixation pins placed at the upper side of the glass bottle. To remove this artifact, we propose to use carbon fixation pins and posts, and this technique will be tested in the future.

One of purposes for the current study was to confirm the validity of the dose prescription method used for treatment plans with multiple matrices for multiple targets. This study (phantom B) had four targets, each enclosed in a dose matrix individually. The prescriptions were 8 Gy to 50.0% to one matrix and 4 Gy to 50.0% for the remaining three matrices. The peak dose of shot #2 in matrix B was measured to be 48.2% of the maximum dose of 16 Gy. Hence, the validity of this prescription method has been demonstrated. For other matrices, the 3D measurements showed, however, that the relative peak doses were 58.2% and 36.4% for shots #3 and #4, respectively. The difference for shot #3, which was an 8 mm collimator beam, may be caused by the 2‐mm‐slice thickness used for the dose measurement. The large error with shot #4 can be partially attributed to the image artifacts due to the fixation pins placed near this shot location. This artifact also caused relatively large differences in the isodose curves between the measurements and calculations, as seen in Fig.5 (f).

In this study, we demonstrated one of unique capabilities of the 3D dosimetry. By inspecting dose distributions on three consecutive transverse planes of Figs. 5 (b), (c), and (d), one could easily identify that the reconnection point at which the measured dose coincides that of the calculated dose in 3D space within 1 mm. It is worth emphasizing that visualization of this reconnection point location in a 3D space is extremely difficult to accomplish with other traditional dosimetry tools such as radiographic films. A practical application of the reconnection point in this case is to use this point as a dose normalization point. By renormalizing the doses for both LGP calculated and measured dose distributions in reference to this point, dose distribution comparision can be accomplished more accurately in a convincing way.

Several authors have shown in their studies that, if there is any dosimeter which can perform comprehensive dosimetry of the whole irradiation exposure, it would be only gel dosimeter.^(^ ^2^ ^,^ ^19^ ^,^ ^20^ ^)^ To verify the same multiple matrices plan as the current study with film dosimetry, several films have to be incorporated into the phantom at different positions. This is more easily said than done. The solid spherical phantom routinely used for Gamma Knife QA allows us to perform film measurement only at limited positions. During Gamma Knife QA, the solid phantom is fixed directly on the couch instead of being set in the Leksell frame. This positioning method always demands that the coordinates be set to be around X=Y=Z=100 mm. In our study, there was more than one Z value (see Table 1); hence, it is impractical to verify dose distributions at those positions using film dosimetry. It should be noted that the dose distribution created by a single collimator is almost spherical in distribution. Even if a plan is made in such a way that the collimator is positioned at X=Y=Z=100 mm, only a planar distribution is obtained by the film dosimetry. To obtain the whole spherical distribution, at least four to five films have to be used and positioning those films is impractical. Taking all these factors into consideration, a simple spherical glass container filled with polymer gel and positioned in the Leksell frame can simulate the whole radiation pattern easily.

## V. CONCLUSIONS

In this study, we accomplished a true 3D dose comparison of LGP calculations and BANG3 polymer gel measurements for a GKSRS plan using multiple shots placed in multiple matrices. The gamma‐index analysis showed an 86% passing rate in a limited region of the measured 3D volume, implying an acceptable agreement between measured and calculated dose distributions. We also showed the agreement of the reconnection point in a 3D space between measurement and calculation. These results demonstrate the value of 3D dose measurements using polymer gel dosimeter. The current work suggests that, with the right analytical tools, the polymer gel dosimetry is not only applicable to GKSRS plans using single shot or multiple shots in single matrix, but also to highly complicated treatment plans such as those with multiple matrices.

## ACKNOWLEDGMENTS

This work was funded by UICC through travel grant fellowship ICRETT No: ICR/08/098. Additional support for this project was given by Atomic Energy Regulatory Board (AERB) through project no N‐964. We would like to gratefully acknowledge their support.
